# Improving cancer incidence evaluation through local government area matching: a study of the Edo-Benin cancer registry in Nigeria

**DOI:** 10.1186/s12889-024-17972-6

**Published:** 2024-02-19

**Authors:** Gregrey A. Oko-oboh, Anssi Auvinen, Darlington E. Obaseki, Janne Pitkäniemi

**Affiliations:** 1 Health Sciences Unit, Faculty of Social Sciences, Tampere University, Arvo Ylpön Katu 34, 33520 Tampere, Finland; 2 Histopathology Department, University of Benin, Benin City, Nigeria; 3 Finnish Cancer Registry, Helsinki, Finland; 4 Department of Public Health, University of Helsinki, Helsinki, Finland

**Keywords:** Cancer registration, Local Government Area, Incidence, Edo-Benin, Nigeria

## Abstract

**Background:**

Cancer registries in Nigeria, as well as in other sub-Saharan African countries, face challenges in adhering to international cancer registration standards. We aimed to improve cancer incidence estimation by identifying under-reporting of new cancers through matching patient-reported local government areas (LGAs) in Edo state, Nigeria, to their respective catchment populations.

**Methods:**

Information on cancers was obtained from records of hospitals, medical clinics, pathology laboratories, and death certificates according to IARC guidelines. We utilized normalized scores to establish consistency in the number of cancers by calendar time, and standardized incidence ratios (SIR) to assess the variation in cancer incidence across LGAs compared to Edo state average. Subsequently, we estimated sex- and site-specific annual incidence using the average number of cancers from 2016 to 2018 and the predicted mid-year population in three LGAs. Age-standardization was performed using the direct method with the World Standard Population of 1966.

**Results:**

The number of incident cancers consistent between 2016–2018 in Egor, Oredo, and Uhunmwonde showed a significantly increased SIR. From 2016 to 2018 in these three LGAs, 1,045 new cancers were reported, with 453 (42.4%) in males and 592 (57.6%) in females. The average annual age-standardized incidence rate (ASR) was 50.6 (95% CI: 45.2 – 56.6) per 10^5^. In men, the highest incidence was prostate cancer (ASR: 22.4 per 10^5^), and in women, it was breast cancer (ASR: 16.5 per 10^5^), and cervical cancer (ASR: 12.0 per 10^5^). Microscopically verified cancers accounted for 98.1%.

**Conclusions:**

We found lower age-standardized incidence rates than those reported earlier for the Edo state population. Collecting information on the local government areas of the cancers allows better matching with the respective target population. We recommend using LGA information to improve the evaluation of population-based cancer incidence in sub-Saharan countries.

## Introduction

Cancer registration began in sub-Saharan Africa (SSA) in the 1950s [1], more than two decades after the first modern cancer registry was established in Europe and the United States [2]. In 2019, the World Health Organization African region (WHO AFRO), reported that 36 of the 47 member countries have cancer registries, and 26 have a population-based cancer registry (PBCR) [3]. In the 2021 report of Cancer Incidence in Five Continents by the International Agency for Research on Cancer (IARC), data were included only from 6 of 21 African countries that submitted data, excluding Nigeria [4]. In these countries, 7 of the 30 cancer registries were accepted, accounting for only 1% of Africa’s population [4].

In Nigeria, cancer registration started with the establishment of the Ibadan cancer registry in 1960 [5]. Currently, there are 33 cancer registries (13 population-based and 20 hospital-based) coordinated centrally by the Nigerian National System of Cancer Registries (NSCR) [6]. The registries are unevenly distributed in the country with two-thirds (22) located in the southern region of Nigeria. All six regions of Nigeria have at least one PBCR, with the South-South (4) and South-West (3) regions having the most. In the IARC GLOBOCAN 2020 publication, data from only four PBCRs in Nigeria (Abuja, Calabar, Ekiti and Ibadan) were included [7]. These four registries cover 3.9% of the Nigerian population based on the catchment areas of the registries [6], and 0.7% of the sub-Saharan African population, even though the population of Nigeria is more than 20% of the entire sub-Saharan Africa population [8].

The Edo-Benin cancer registry (EBCR) was founded in 2008 as a hospital-based cancer registry (HBCR). In 2015, it was designated as a PBCR. Currently, it is one of the 13 PBCRs in Nigeria [6], and data from EBCR were used in conjunction with another PBCR to derive the most recent national cancer incidence estimates for Nigeria by the Nigerian National Systems for Cancer registries (NSCR) [6].

IARC, in its scientific publication No. 95, suggested that ‘developing countries should define registry areas using administrative boundaries (in the case of Nigeria; local government areas) which can be matched both with the patient addresses and data on the size of the population at risk, typically derived from census information’ [2]. As far as we are aware, no study has employed this recommended method for estimating cancer incidence in sub-Saharan Africa (SSA).

In Nigeria, the catchment areas of PBCRs are not clearly defined. Consequently, catchment areas are usually chosen based on the location of PBCRs or referral health facilities, primarily within urban areas [1, 9]. However, the cancers documented by these registries often originate from the entire sub-national region or even beyond [1]. Therefore, there is a pressing need to match reported cancers with their respective catchment areas to identify areas of under-reporting within the sub-region and to enhance the accurate evaluation of cancer burden.

Our primary objective in this study was to accurately estimate cancer incidence in Edo-Benin. Our secondary objective was to identify underreporting, which was accomplished by matching patient-reported Local Government Areas (LGAs) to their respective catchment populations. Additionally, we evaluated the quality of cancer registration.

## Methods

### Edo state and cancer care facilities

**Fig. 1:**
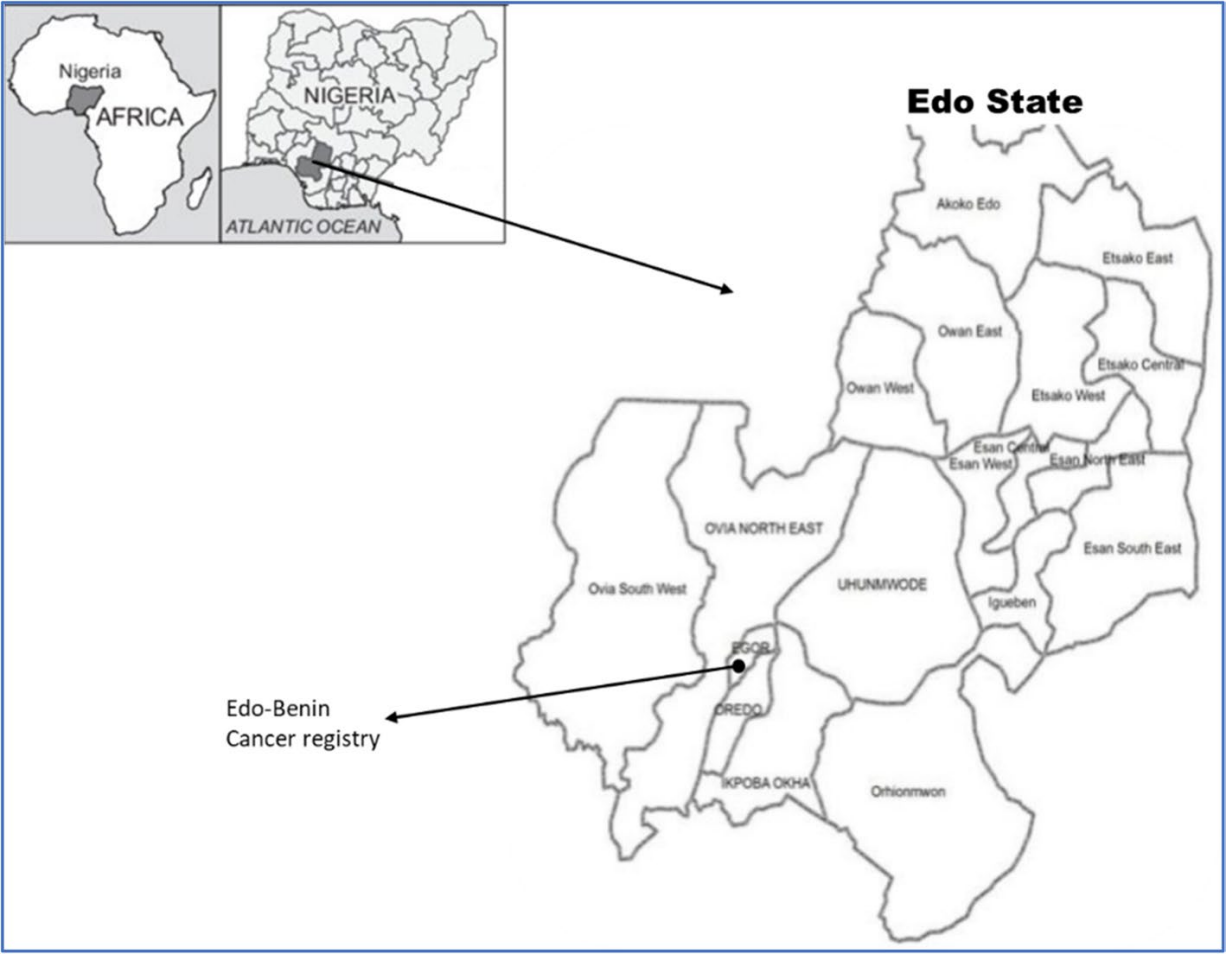
Map of Edo State, its 18 Local Government Areas, and Edo-Benin cancer registry

Edo state is in the South-South geopolitical zone of Nigeria, with its capital at Benin City. It has an estimated population of 3,218,332 (1,640,461 males and 1,577,871 females) spread across 18 local government areas (LGAs) [10]. The state accounts for 2.3% of Nigeria’s population (Fig. 1).

The University of Benin Teaching Hospital (UBTH) is the only facility providing specialized cancer care services [11]. It serves as a major referral center for suspected cancers cases from other health facilities, both within and outside Edo state. The Edo-Benin Cancer Registry is located at the UBTH [12].

### Case finding

Information on cancer cases was manually abstracted by cancer registrars trained by the Nigerian National Systems of Cancer Registries (NSCR). They visited six facilities weekly. These healthcare facilities include the publicly funded University of Benin Teaching Hospital (tertiary health facility) and Central Hospital (secondary health facility), as well as the privately funded Faith Medical Complex and Saint Philomena Hospital (both secondary health facilities). Additionally, the diagnostic laboratories, Biogenics Pathology and Asamah Foundation, both operate with private funding. For each cancer case, patient demographic and clinical information was recorded, including topography, morphology, histology, grade, basis of diagnosis and treatment. The abstracted cancer data was then enteredinto the CanReg5 software, following IARC guidelines for cancer information production. Cancers were identified using patient name, sex, birth date/ age and diagnosis. Topography, morphology and behaviour of abstracted cancers are coded using ICD-O-3 (C00 to C95), which was later converted to ICD-10 for statistical tabulation.

Multiple primaries were coded according to the rules jointly developed by the International Association of Cancer Registries (IACR) and the IARC [13]. Death certificates were actively sought from mortuaries. Conflicting cancer information between pathological and clinical data was resolved through a comprehensive review of the initial biopsy or surgical specimen by a pathologist, in addition to a clinical review to form a consensus. The date of diagnosis was recorded as the first admission date to any of the participating six health facilities. The date was extracted from all available medical records with suspected cancer. We considered cancer as microscopically verified (MV) if the basis of diagnosis was cytology or histology, either from primary, metastases, or autopsy.

### Study variables

The independent variables included age, sex, local government areas (LGAs) in Edo state, and reporting years (spanning from 2009 to 2018). Age in years was categorized into eight groups (0–14, 15–24, 25–34, 35–44, 45–54, 55–64, 65–74, 75 and above), while sex (male, female) and LGAs are categorical variables with nominal scale measurements. The LGAs are the various local administrative units in Edo State. The reporting years, spanning from 2009 to 2018, are treated as discrete variables.

The dependent variables encompass the number of cancers (which is measured as discrete nominal), and cancer types and their classifications. Cancer types are categorical variables with nominal scale measurements. The cancers were categorized into 23 males and 27 females’ cancer sites based on the ICD-10 guidelines.

### Statistical methods

To estimate changes in the annual reporting of cancers, we used Poisson regression with the expected number of cancers as outcome. We compared the expected annual number of cancers between 2011 and 2018 to the average number of cancers in 2009 and 2010. The case finding in 2009 and 2010 was well organized and resourced by personnel and thus considered to be as complete as possible [14]. Furthermore, cuts in personnel after 2010 led to inability to collect and process pathological and clinical reports as before. We considered years with p-values less than 5% as outliers (indicating underreporting).

To estimate changes in the annual reporting of cancers, we used poisson regression as a statistical method for time series analysis, with the expected number of annual cancers as outcome. We compared the expected annual number of cancers between 2011 and 2018 to the average number of cancers in 2009 and 2010 was used as reference. This choice of reference was based on the case finding in 2009 and 2010 was well organized and resourced by personnel and thus considered to be as complete as possible. We consider years with p-values less than 5% as outliers (indicating underreporting).

We reported the relative levels of cancer incidence by local government areas using standardized incidence ratios (SIR), with the total cancer incidence for all 15 LGAs in Edo-Benin as the reference. For each LGA and gender, we calculated the observed and expected numbers of cancers across five-year age categories (0–4, 5–9,…,80–84, and 85 +) and one-year calendar time periods (2016, 2017, and 2018). The SIR was then calculated as the ratio of the observed to expected cancer cases. For each SIR, we calculated the exact 95% confidence interval (CI), assuming a Poisson distribution of the observed number of cases. If the SIR lower limit was above 1, implying better than average statewide registration. These were considered reliable for the estimation of cancer incidence.

After excluding cancers due to annual under-reporting and restricting them to three catchment areas, we presented the number of incident cancers for males and females across eight age groups and 23 (males) and 27 (females) cancer sites. Crude incidence rates per 100,000 per year were computed, as well as age standardized rates (ASRs). Age-standardization was done by the direct method using the World Standard Population of 1966 [15, 16]. Statistical data analysis was conducted using the R software version 4.1.2 and the PopEpi package version 0.4.9 (for age-standardized incidence) [17]. We assessed diagnostic validity by examining the percentage of morphologically verified site and sex-specific cancers [15].

### Population

**Fig. 2:**
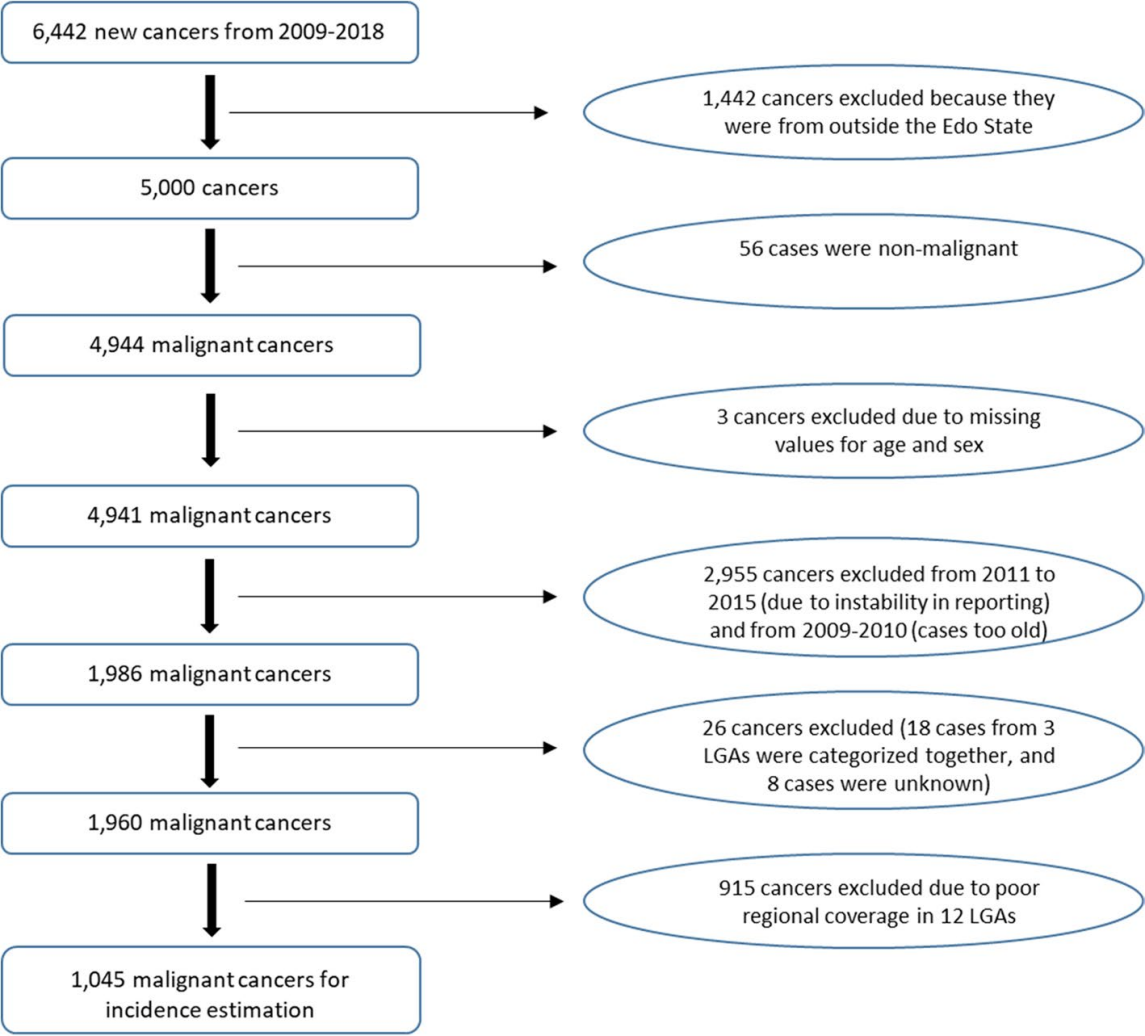
Estimated percentages of population of Benin City in 2017 by age group and sex

Age- and sex- specific populations in Edo, categorized by local government areas (LGAs), were derived from the Nigerian census of 2006 [18], with the annual growth rate applied from the World Bank Statistics [19]. The same annual growth rate was applied for males, females and all age groups. The ASR was estimated using the population at risk within five-year age groups in 2017 (Fig. 2).

### Ethics statement

The ethical issues of this project were reviewed and approved by the Health Research Ethics Committee of the University of Benin Teaching Hospital (Ethical clearance Number: ADM/E 22/A/VOL.V11/148301102). To address privacy concerns, stringent measures were implemented to safeguard the confidentiality of the data. Our study involved the analysis of de-identified data obtained from Edo-Benin Cancer Registry. This data source is a comprehensive and established cancer registry that collects and maintains information on cancer cases in the Edo State population. The data provided to us for analysis were fully anonymized, and no personally identifiable information was accessible to our research team. As a result, our study did not involve direct contact with human participants. Moreover, access to the data was restricted to authorized personnel directly involved in the research, and the data storage complied with industry-standard security protocols.

## Results

**Fig. 3:**
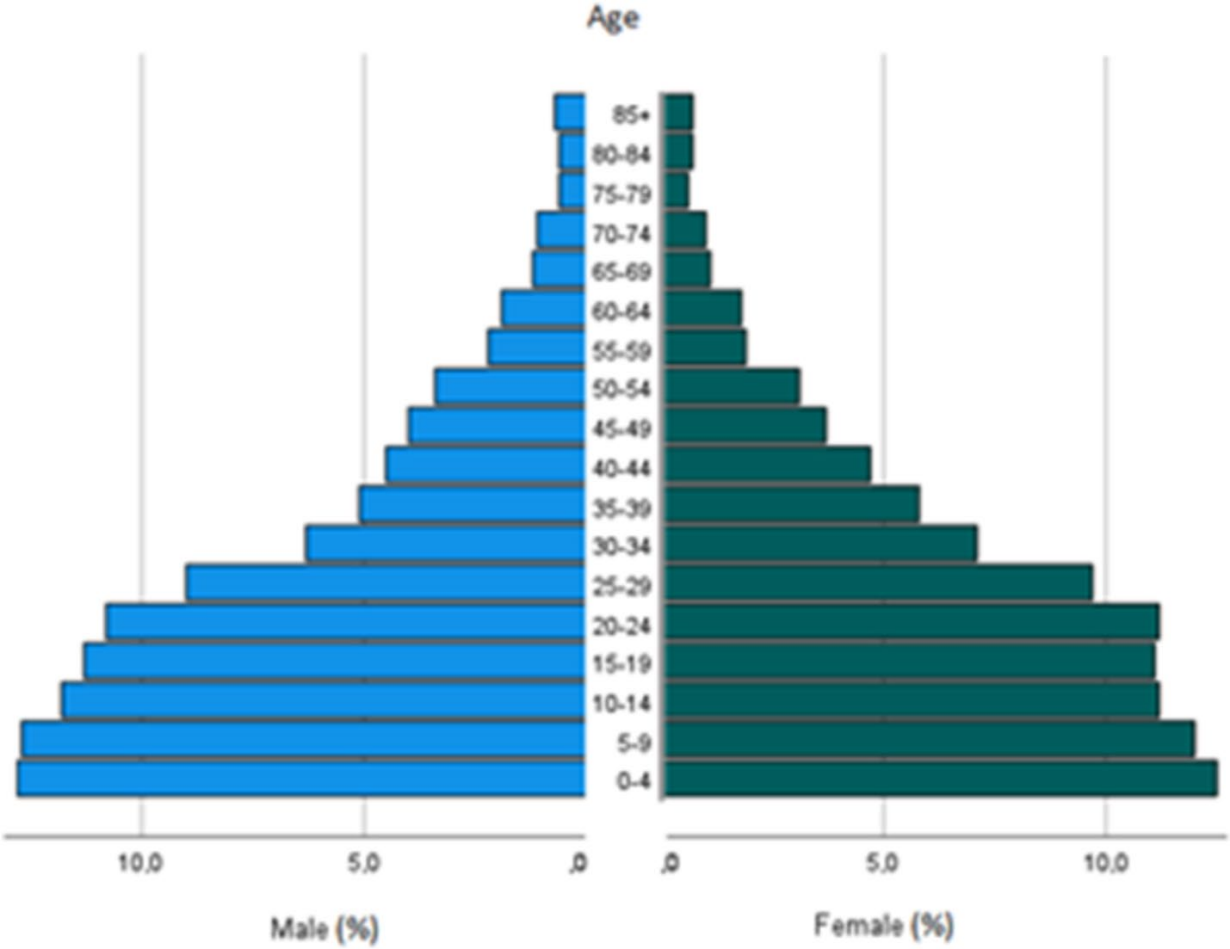
Flow chart of the exclusion criteria for the reported cancers in Edo-Benin cancer registry

A total of 6,442 new cancers were reported to EBCR during the period from 2009 to 2018. Cancers for three LGAs (Owan West, Owan East, and Akoko-Edo) were not separated in the registration and are therefore excluded from the analysis (*n* = 18, Fig. 3.)

Out of the 6,442 new cancers obtained, 1,501 (23.3%) were excluded for the following reasons: 1,442 (22.4%) cancers cases resided outside Edo State at the time of diagnosis, 56 (0.9%) were non-malignant, and 3 (0.05%) had missing values for sex or age (Fig. 3).

### Annual cancer registration and regional variation in cancer incidence

The annual numbers and percentages of cancers by sex for the period 2009 and 2018, obtained by Edo-Benin cancer registry, are shown in Table 1. The reported number of cancers from 2011 to 2015 was lower than the average number of cancers in 2009 and 2010. The number of cancers reported in 2016 returned to pre-2011 numbers and was not statistically significantly different in 2017 and 2018. Because the annual number of cancers from 2011 to 2015 indicated underreporting, cancers from these years were excluded, and we reported the most recent years of 2016 to 2018 (Fig. 3).

**Table 1:** Numbers of cancers and percentages by year of diagnosis and sex in all local government areas of Edo-Benin

Year of diagnosis	Male N (%)	Female N (%)	Total N (%)
2009	341 (12)	228 (11)	569 (11.5)
2010	464 (16)	319 (16)	783 (15.8)
2011	203 (7.0)	153 (7.5)	356 (7.2) *
2012	99 (3.4)	66 (3.2)	165 (3.3) *
2013	197 (6.8)	153 (7.5)	350 (7.1) *
2014	223 (7.7)	157 (7.7)	380 (7.7) *
2015	224 (7.7)	128 (6.3)	352 (7.1) *
2016	463 (15.9)	265 (13)	728 (14.7)
2017	339 (12)	267 (13)	606 (12.3)
2018	356 (12)	296 (15)	652 (13.3)
Total	2909 (58.9)	2032 (41.1)	4941 (100.0)

^*^ Significantly lower (*p* < 0.05) annual numbers of cancers compared to average number of cancers in 2009 and 2010 with Poisson regression of the annual expected number of cancers

The number of cancers and standardized incidence ratios (SIR) with 95% confidence intervals (CI) for the 15 LGAs in Edo-Benin are shown in Table 2. Seven LGAs had SIRs less than Edo state average (Esan North-East, Ikpoba-Okha, Etsako West, Esan South East, Ovia North East, Etsako East, Ovia South West). The lowest SIRs were in Esan-South East (0.17, 95% CI: 0.11–0.25) and Etsako East (0.26, 95% CI: 0.18–0.37). Five LGAs (Orhionmwon, Esan West, Esan Central, Etsako Central and Igueben) had SIRs corresponding to the state average incidence. Standandised incidence ratios were statistically significantly for Egor, Oredo, and Uhunmwonde. The standardized incidence of Egor (1.96, 95% CI: 1.79–2.15) was almost twice as high as Edo state average and in both Oredo (1.65, 95% CI: 1.50–1.81) and Uhunmwonde (1.57, 95% CI: 1.34–1.82) there was more than a 50% increase in incidence. We restricted the analysis to these three LGAs because they are least likely to contain underreporting of cancers.

**Table 2:** Overall populations, number of cancers and standardized incidence ratios (SIR) with 95% confidence intervals (CI) by local government areas (LGAs) in Edo-Benin

LGAs	Population	Cancers	SIR (95% CI)
Oredo	374,515	440	1.65 (1.50–1.81)
Ikpoba-Okha	372,080	179	0.72 (0.62–0.83)
Egor	340,287	445	1.96 (1.79–2.15)
Etsako West	198,975	58	0.41 (0.31–0.52)
Orhionmwon	183,994	164	1.07 (0.91–1.24)
Esan South-East	166,309	21	0.17 (0.11–0.25)
Ovia North-East	155,344	77	0.72 (0.57–0.89)
Etsako East	147,335	29	0.26 (0.18–0.37)
Ovia South-West	138,072	47	0.51 (0.37–0.66)
Esan West	127,718	80	0.85 (0.68–1.05)
Esan North-East	121,989	43	0.46 (0.33–0.61)
Uhunmwonde	121,749	160	1.57 (1.34–1.82)
Esan Central	105,242	76	0.93 (0.73–1.15)
Etsako Central	94,228	76	1.09 (0.87–1.36)
Igueben	70,276	65	1.14 (0.89–1.44)
**Total**		**1960**	

### Cancer incidence in Edo State

A total of 1,045 incident cancers were reported [453 (42.4%) in men and 597 (57.6%) in women] from 2016 to 2018 in three LGAs (Egor, Oredo, and Uhunmwonde), in which the SIRs were above the Edo-Benin average (Table 3). The average annual number of incident cancers was 350 (151 in men and 199 in women) with an average annual ASR of 50.6 (95% CI; 45.2 – 56.6) per 100,000. The average annual ASR was 47.4 (95% CI; 40.1 – 56.1) per 100,000 for males and 56.0 (95% CI; 48.2 – 65.0) per 100,000 for females. The ASR for all cancers within Edo State in the registry for 2016–2018 restricted to three LGAs (unmatched) was 94.8 (95% CI: 87.3 – 102.8).

**Table 3:** Population, number of cancers, crude, and age-standardized incidence of Edo- Benin Cancer Registry from 2016 – 2018 in three LGAs

	**Total**	**Male**	**Female**
Population size	1,138,070	573,484	564,586
Cancers	1,045	453	592
Av. annual number of cancers	348	151	197
Average crude rate (95% CI) ^a^	30.7 (27.6 – 34.1)	26.3 (22.4 – 30.9)	35.2 (30.7 – 40.5)
Average ASR (95% CI) ^a^	50.6 (45.2 – 56.6)	47.4 (40.1 – 56.1)	56.0 (48.2 – 65.0)

ASR for all cancers within Edo State in the registry for 2016–2018 restricted to three LGAs (unmatched) was 94.8 (95% CI: 87.3 – 102.8)

^a^per 10^5^, ASR; age-standardized rate, LGAs included: Egor, Oredo, and Uhunmwonde

The number of incident cancers, crude, and age standardized incidence of primary cancers by sex, site, and age group in EBCR are shown for males in Table 4 and females in Table 5. The most common cancers (average annual number, percentage) in males were prostate (343, 58.5%), colon (27, 4.4%), rectum (19, 3.8%), and pharynx (18, 3.1%). The age-standardized incidence rate for prostate was 29.2 per 100,000, colon 1.9 per 100,000, rectum 1.3 per 100,000, and pharynx 1.2 per 100,000. The average annual numbers of prostate cancers were substantially higher at ages 45–54 years compared with 35–44 years (22 vs 3). A similar increase was also observed across other age groups. In males, the non-solid cancers (C81 – C96) accounted for 2.4% of the incident cancers, with non-Hodgkin lymphoma (1.5%) and Hodgkin lymphoma (0.9%) as the most common non-solid cancers. The ‘other malignant neoplasms of skin’ (C44) accounted for 2.0% of all incident cancers in males.

**Table 4:** Site and age specific number of cancers and site specific morphologically verified, crude rates and age-standardized rates for males, 2016–2018

Site	ICD-10	0–14	15–24	25–34	35–44	45–54	55–64	65–74	75 +	Sum	%	MV%	CR	ASR
Pharynx	C9-11, C12-14	1	1	0	1	3	6	2	0	14	3.1	100	0.7	1.2
Oesophagus	C15	0	0	0	1	0	2	2	2	7	1.5	100	0.2	0.4
Stomach	C16	0	0	0	1	0	3	2	0	6	1.3	100	0.2	0.3
Colon	C18	0	0	1	1	3	4	7	4	20	4.4	96.3	1	1.9
Rectum	C19-20	0	0	1	4	3	2	6	1	17	3.8	94.7	0.7	1.3
Anus	C21	0	0	0	1	0	1	0	0	2	0.4	100	0.0	0.0
Liver	C22	0	0	0	0	2	1	0	0	3	0.7	100	0.0	0.0
Pancrease	C25	0	0	0	0	1	0	0	0	1	0.2	100	0.0	0.0
Trachea bronchus lung	C33-34	0	0	0	3	3	1	2	0	9	2.0	91.7	0.3	0.5
Melanoma of skin	C43	0	0	0	0	0	1	0	0	1	0.2	100	0.0	0.0
Other skin	C44	0	1	2	2	3	1	0	0	9	2.0	100	0.2	0.3
Kaposi’s sarcoma	C46	0	0	0	1	0	0	0	0	1	0.2	100	0.0	0.0
Connective soft tissue	C49	1	0	0	0	2	0	1	2	6	1.3	100	0.3	0.4
Breast	C50	0	0	0	3	1	6	1	1	12	2.6	91.7	0.5	0.9
Prostate	C61	0	1	1	3	22	55	87	96	265	58.5	98.5	15.2	29.2
Kidney	C64-65	0	0	0	1	0	0	1	0	2	0.4	100	0.0	0.0
Ureter bladder	C66-68	0	0	0	1	1	1	1	1	5	1.4	100	0.2	0.3
Thyroid	C73	0	1	0	0	1	0	0	0	2	0.4	100	0.0	0.0
ill-defined, secondary and unspecified sites	C76, C80	0	1	0	1	2	4	2	0	10	2.2	100	0.3	0.7
Hodgkin lymphoma	C81	0	0	0	2	1	0	1	0	4	0.9	100	0.2	0.2
Non-Hodgkin lymphoma	C82-85, C96	1	2	2	0	1	0	1	0	7	1.5	100	0.2	0.1
Multiple myeloma	C90	0	0	1	0	0	0	0	0	1	0.2	100	0.0	0.0
Other and unspecified	Other	3	2	1	11	10	8	12	2	49	10.8	98.3	2.6	4.4
All sites total		6	9	9	37	59	96	128	109	453	100	98.3		
% total		1.3	2	2	8.1	13	21.2	28.3	24.1	100				

Unclassified cancers in males included ICD-10 codes; C17, C23, C26, C30, C38, C41, C62, C63, C69, C77, and C86

**Table 5:** Site- and age-specific number of cancers and site specific morphologically verified, crude rates and age-standardized rates for females, 2016–2018

Site	ICD-10	0–14	15–24	25–34	35–44	45–54	55–64	65–74	75 +	Sum	%	MV%	CR	ASR
Pharynx	C9-11, C12-14	0	0	0	1	3	1	0	0	5	0.8	100	0.2	0.1
Oesophagus	C15	0	1	0	0	1	1	5	0	8	1.4	100	0.2	0.5
Stomach	C16	0	1	1	2	3	1	3	1	12	2	93.3	0.5	0.8
Colon	C18	0	0	1	2	3	6	0	2	14	2.4	94.7	0.7	1.4
Rectum	C19, C20	0	0	1	4	2	2	1	1	11	1.9	100	0.5	0.8
Anus	C21	0	0	0	0	2	1	3	0	6	1	100	0.2	0.4
Liver	C22	0	0	0	1	0	1	1	1	4	0.7	100	0	0
Pancrease	C25	0	0	1	0	0	2	1	0	4	0.7	80.0	0.2	0.4
Larynx	C32	0	0	0	1	0	0	1	0	2	0.3	100	0	0
Trachea bronchus lung	C33, C34	0	0	2	2	1	2	4	1	12	2	100	0.7	1.2
Melanoma of skin	C43	0	0	0	0	0	1	0	1	2	0.3	100	0	0
Other skin	C44	0	1	1	4	3	3	0	0	12	2	100	0.5	1
Connective soft tissue	C49	0	0	0	0	2	1	1	1	5	0.8	100	0.2	0.3
Breast	C50	0	2	19	52	64	41	25	9	212	35.8	97.9	12.4	18.8
Vagina	C52	0	0	2	2	2	2	1	2	11	1.9	100	0.4	0.3
Cervix uteri	C53	0	1	5	27	42	42	21	9	147	24.8	98.5	8.9	14.5
Corpus uteri	C54	0	0	0	3	6	0	3	0	12	2	100	0.7	0.9
Uterus unspecified	C55	0	0	0	5	6	7	2	0	20	3.4	92.0	1.2	2.3
Ovary	C56	0	3	4	8	9	3	3	0	30	5.1	100	1.8	2.1
Kidney	C64, C65	0	0	0	1	0	0	0	0	1	0.2	100	0	0
Ureter Bladder	C66-68	0	0	0	1	1	1	0	2	5	0.8	100	0	0
Brain nervous system	C70-72	0	0	0	0	1	0	3	0	4	0.7	75.0	0.2	0.5
Thyroid	C73	0	0	2	3	2	0	0	0	7	1.2	100	0.4	0.5
ill-defined, secondary and unspecified sites	C76,C80	0	0	0	1	2	1	2	0	6	1	100	0.2	0.3
Hodgkin lymphoma	C81	0	1	0	1	0	0	0	0	2	0.3	100	0	0
Non-Hodgkin lymphoma	C82-85, C96	1	1	0	0	0	1	0	0	3	0.5	100	0	0
Other and unspecified	Other	3	1	1	3	5	7	13	2	35	5.9	95.7	2.1	3.8
All sites total		4	12	40	124	160	127	93	32	592	100	97.9		
% total		0.7	2	6.8	20.9	27	21.5	15.7	5.4	100				

Unclassified cancers in males included ICD-10 codes; C17, C23, C26, C38, C40, C41, C48, C51, C69, C75, C77, and C86

In females, the most common cancers (average annual number, percentage) were breast (212, 35.8%), cervix uteri (147, 24.8%), and ovary (30, 5.1%). The ASR for these sites were breast 18.8 per 100,000, cervix uteri 14.5 per 100,000, and ovary 12.1 per 100,000.

The proportion of breast cancers peaked in the age group 45–54 years (30.2%). Non-solid cancers (C81 – C96) accounted for 0.8% of total reported cancers in females. ‘Other malignant neoplasms of skin’ (C44) accounted for 2.0%.

There were 10 incident childhood cancers accounting for 1.0% of all incident cancers (1.3% in males, and 0.7% in females). The most common type of childhood cancer was non-Hodgkin lymphoma (2 cancers) and half (6 cancers) of the childhood cancers were defined as ‘other and unspecified’.

Three-fourths (73.5%) of the cancers were diagnosed in individuals aged 55 years or older in males. Additionally, 46.4% of cancers were in the working age (15 – 64 years) population, and 52.3% among the elderly (65 years and above) population. The most reported cancers in the working-age population in males were prostate (39.0%), colon (4.3%), and pharynx (5.2). In females, 78.2% of the cancers occurred at the working-age (15 – 64 years), and 21.1% occurred in the elderly (65 years and above). Incidence was highest in the age group 45–54 years in females.

The percentage of microscopically verified cancers was 98.1% (98.3% in males and 97.9% in females). All sites had 100% morphological verification in males, except for prostate (98.5%), colon (96.3%), rectum (94.7%), breast (91.7%) and trachea, bronchus, lungs (91.7%). A Lower proportion of morphologically verified cancers was observed in females with seven cancers having morphological verification less than 100%: cervix uteri (98.5%), breast (97.9%), colon (94.7%), stomach (93.3%), uterus unspecified (92.0%), pancreas (80.0%) and brain, nervous system (75.0%).

## Discussion

The use of local government area (LGA) information commonly reported by patients in cancer registration in Nigeria, together with matching target population, should be used for more reliable estimation of cancer incidence. This is important when cancer registration does not adequately cover the entire target population (state). When cancer and population data were matched, the age-standardized cancer incidence of Edo-Benin was 51/100,000 per year, with a lower incidence in men (47/100,000) than in women (56/100,000). The most common cancers were prostate, breast and cervix cancers.

The age-standardized cancer incidence reported by GLOBACAN in 2020, using cancers from four Nigerian population-based cancer registries (Abuja, Ekiti, Calabar, and Ibadan), was 110/100,000 per year [7]. This rate is more than two-fold higher than the cancer incidence estimate for Edo-Benin. The Nigerian consortium estimated the average annual age-standardized incidence of EBCR in 2015 as 88/100,000 [6]. This figure is almost twice as high as what we evaluated for Edo-Benin.

If we were to include all cancers within Edo State reported in the registry and restrict the target populations to three LGAs (without matching, as was the earlier practice) from 2016 to 2018, then the average annual ASR for the Edo-Benin cancer registry can be estimated as 95/100,000 per year (95% CI, 87.3 – 102.8). This is closer to the figures reported by GLOBOCAN and the Nigerian consortium, which is consistent with the proposition that population-based cancer registries (PBCR) in Nigeria have not matched cancers with proper catchment areas. Catchment areas are typically selected to be the urban areas, where the PBCR or referral health facilities are located [1, 19], while the cancers in these registries come from the entire sub-national region or even outside the region [1]. Matching reported cancers and their catchment area by LGA is needed to obtain a more accurate assessment of cancer incidence, and the incidence reported by GLOBOCAN, and the Nigerian consortium potentially may have overestimated the true incidence for Nigeria.

Our approach addressed the main challenges in cancer surveillance in sub-Saharan Africa related to assessment (clear definition) of the catchment population of PBCR and the availability of population denominators (lack of census data). LGAs are the smallest sub-national and geographical units for defining residence in Nigeria. It also forms the bedrock of the primary health care system in Nigerian and Edo State [20]. While PHC centers are built in each LGA, the Specialist and Teaching hospitals where cancer care is provided do not follow this pattern. Rather, in Edo State, the teaching hospital (UBTH) providing oncological services and housing the cancer registry is built in the urban LGA. Suspected cancers residing in or close to LGAs where UBTH is located are more likely to seek care at the teaching hospital and thus more likely to be captured by the cancer registration system. We computed a more valid estimate of cancer incidence by restricting the population at risk to LGAs that match the place of residence of cancer cases at time of diagnosis. The IARC guidelines also recommended the use of local government population as denominators in the computation of cancer incidence in places where census data are not available and where registry catchment definition is not formally and geographically defined [2]. Our method of comparing relative proportions of cancers to those expected by population sizes by LGA relies on the fact that differences reflect mainly differences in registration. Such differences can be caused by differences in risk factors between LGAs, but the main reason is likely the care-seeking pattern favoring suspected cancers nearby UBTH.

In men, the highest age-standardized incidence rates in Edo-Benin were for prostate cancer. The GLOBOCAN 2020 rates for Nigeria also reported prostate cancer as the most common cancer for men [7]. However, the GLOBOCAN rates were higher than those of our findings (35 vs. 29). Prostate cancer is the most frequent (58%) cancer in men in Edo-Benin. This is consistent with previous studies reporting prostate cancer as the most common cancer in Nigerian men (between 32 and 46% by other Nigerian registries) [21]. In 2020, GLOBOCAN estimated that prostate cancer accounted for 30% of all male cancers in Nigeria [7]. The high proportion of prostate cancer in Edo-Benin could be attributed to broader use of the prostate specific antigen (PSA) due to active recruitment.

Our study also reported an increase in the number of prostate cancers with an increase in age. The risk of developing prostate cancer increased significantly after the age of 50 years, with more than 80% of prostate cancers diagnosed in men over the age of 65 years [9, 22]. The age-related increase in prostate cancer incidence is particularly relevant for Nigeria, where the population is projected to age rapidly in the coming years. According to the United Nations, the proportion of Nigerians aged 65 years or older is expected to increase from 3% in 2020 to 6% in 2050 [23]. This demographic shift could result in a significant increase in the number of prostate cancer cases in Nigeria.

Similar to men, the incidence rates for the most common cancers in females were lower than those in GLOBCAN 2020. Compared to the Edo-Benin cancer registry, GLOBOCAN 2020 reported higher age-standardized rates (per 100,000) for breast cancer (49.0 vs 18.8), cervical cancer (18.4 vs 14.5), and ovarian cancer (5.6 vs 2.1). Yet, there were less differences in ranking by primary site. The most common cancers were breast (35.8%), cervix uteri (24.8%), and ovarian (5.1%). Likewise, GLOBOCAN 2020 reported breast (38.7%), cervix uteri (16.4%), non-Hodgkin lymphoma (4.8%), and ovary (4.4%) as the most common cancers. The percentage of breast cancer and ovarian cancer is similar to GLOBOCAN.

Childhood cancers accounted for 1.0% of all incident cancers in EBCR, with non-Hodgkin lymphoma and connective soft tissue cancers being the most common types. Earlier reports from the Edo-Benin cancer registry (2014 – 2016) show similar proportion of 1.4% [6]. In contrast, GLOBOCAN 2020 reported a higher proportion of 5.3%, with leukaemia, brain/central nervous system, and non-Hodgkin lymphoma being the most common types [24]. The lower rates of childhood cancers in the EBCR than GLOBOCAN are likely due to underreporting [16, 25].

The overall percentage of morphologically verified cancers was high (98%) for a developing country [26] and high when compared with other registries in Nigeria [14]. This high value could reflect the type of facilities reporting data to the EBCR. Two of the facilities contributing data to the registry are pathology laboratories, and the host institution (that is, UBTH) of the EBCR has a fully functional pathology department. Thus, information on most cancers in the EBCR would include a histological report. The MV% was similar in males (98.3%) and females (97.9%). For males, all cancer sites had a morphological verification of 100%, except for prostate, colon, rectum, breast, and lung. In females, the pancreas (80%), and brain/nervous system (75%) had MV% far less than 100%. A high percentage of morphological verification is necessary for validity, but extremely high levels may suggest a reliance on the pathology laboratory as the only source of information. This may reflect a failure to detect cases diagnosed by other means and result in incomplete registration [15]. In Nigerian cancer registries in 2015, low MV% for cancers were noted [14]. The lower numbers may be due to late-stage diagnosis of these malignancies, which would make them less suitable for biopsy and surgical treatment.

The inconsistencies in the yearly reported numbers of cancers and case registration practices in the EBCR show the need for validation studies for quality assurance and further development of cancer registration. The drop in reported numbers of cancers coincided with a change in the management of the cancer registration in Edo-Benin in 2010 [6]. Our results also show a substantial improvement from 2016 due to the designation of the EBCR as PBCR in 2015.

We acknowledge the inherent limitations of relying on self-reported information for residence determination used in this study. However, in the absence of more precise data sources, such as comprehensive address databases, we utilized the best available information provided by the patients themselves which is routinely collected by the cancer registry during data abstraction. We believe that our approach, though not perfect, represents a reasonable and practical strategy given the data constraints in the context of our study.

Furthermore, the low death registration levels in Nigeria, estimated at only 10% [27], significantly compromise the validity of cancer data in the country. This limitation is closely linked to missed cases of cancers in cancer registration, as a significant portion of deaths goes unregistered. The cause of death remains uncertain for many individuals, making it challenging to accurately attribute deaths to specific cancers. This lack of vital information results in underreporting of cancer-related deaths in cancer registries, impacting the overall quality and validity of cancer data. Missed cases and underreporting can lead to an incomplete picture of the cancer burden, hindering comprehensive cancer control efforts, early detection, and effective healthcare planning in Nigeria. Addressing the issue of low death registration is essential for improving the quality and completeness of cancer data.

The findings of this study have significant public health implications. Firstly, we strongly recommend the documentation of the local administrative district of patients at the time of cancer diagnosis, emphasizing its usefulness in estimating accurately cancer incidence. Our study, which involved matching cancers to their respective catchment areas, revealed lower estimates than previously reported, suggesting that earlier cancer incidences from this region were likely overestimated.

Additionally, our analysis indicated that if the major health facilities and cancer registry are within the same local government area these leads to better coverage compared to when they are not in the same LGA. Because of incomplete reporting in many LGAs in Edo-Benin, we recommend the use of our method (to match cancers and populations by LGAs), to get as close-as possible population-based estimates of incidence. Finally, as cancer is not included in the integrated diseases surveillance and response (IDSR) system of Nigeria, we recommend that cancers should be part of the system.

## Conclusions

We found lower age-standardized incidence rates than those previously reported for the Edo state population. Collecting information on the local government areas of the cancers enabled better matching with the respective target population. We recommend incorporating local administrative units (such as LGAs, municipalities, commune, county, etc.) information to enhance the evaluation of population-based cancer incidence in sub-Saharan countries.

## Data Availability

The cancer information provided by the Edo-Benin Cancer registry is only to be used by us. And there is a prohibition on further use and disclosure of this information. All other data that support the findings of this study are available from the corresponding author upon reasonable request.

## Abbreviations

ASR: Age-standardized Incidence Rate
C: Cancer
CI: Confidence Interval
CR: Crude Rate
EBCR: Edo-Benin Cancer registry
GLOBOCAN: Global Cancer Statistics
HBCR: Hospital-Based Cancer Registry
ICD-10: International Classification of Diseases, 10th Revision
ICD-O-3: International Classification of Diseases for Oncology, 3rd Edition
IACR: International Association of Cancer Registries
IARC: International Agency for Research on Cancer
LGA: Local Government Area
MV: Microscopically Verified
NCSR: Nigerian National Systems for Cancer registries
PBCR: Population-Based Cancer Registry
PHC: Primary Health Care
PSA: Prostate Specific Antigen
SIR: Standardized Incidence Ratio
SSA: Sub-Saharan Africa
UBTH: University of Benin Teaching Hospital
WHO AFRO: World Health Organization African Region

## References

[1] Parkin DM, Ferlay J, Jemal A, Borok M, Manraj SS, N’Da GG, et al. Cancer in Sub-Saharan Africa. 2018. Available from: http://publications.iarc.fr/Book-And-Report-Series/Iarc-Scientific-Publications/Cancer-In-Sub-Saharan-Africa-2018. Cited 2022 Apr 22.

[2] Jensen OM, Parkin DM, MacLennan R, Muir CS, Skeet RG, editors. Cancer registration: principles and methods. Lyon, France : New York: International Agency for Research on Cancer, World Health Organization; 1991.

[3] World Health Organization. Fiscal policies to promote healthy diets: policy brief. Geneva: World Health Organization; 2022. Available from: https://apps.who.int/iris/handle/10665/355965. Cited 2022 Oct 24.

[4] Bray F, Colombet M, Mery L, Piñeros M, Znaor A, Zanetti R, Ferlay J, editors. Cancer Incidence in Five Continents, Vol. XI. IARC Scientific Publication No. 166. Lyon, France: International Agency for Research on Cancer; 2021. Available from: https://publications.iarc.fr/597. Licence: CC BY-NC-ND 3.0 IGO

[5] Jedy-Agba EE, Oga EA, Odutola M, Abdullahi YM, Popoola A, Achara P, et al. Developing National Cancer Registration in Developing Countries – Case Study of the Nigerian National System of Cancer Registries. Front Public Health. 2015;3. Available from: http://journal.frontiersin.org/Article/10.3389/fpubh.2015.00186/abstract. Cited 2023 Mar 8. 10.3389/fpubh.2015.00186 PMC4519655 26284233

[6] Akintola A, Odutola M, Olayinka T, Akinjola A, Nwokwu U, Adebamowo C, editors. Cancer in Nigeria 2009 - 2016. 2nd ed. Nigeria: Nigerian National System of Cancer Registries Federal Ministry of Health of Nigeria; 2021. Available from: https://nigeriancancerregistries.net/wp-content/uploads/2021/03/CANCER-IN-NIGERIA-VOLUME-II.pdf. Cited 2022 Apr 25. 36327355

[7] International Agency for Research on Cancer W. IARC Globocan 2020: Nigeria Fact Sheets. IARC GLOBOCAN 2020. Lyon, France: IARC, WHO;p. 2. Available from: https://gco.iarc.fr/today/data/factsheets/populations/566-nigeria-fact-sheets.pdf. Cited 2022 Apr 25.

[8] United Nations, Department of Economic and Social Affairs, Population Division. World Population Prospects 2019, Volume II: Demographic Profiles (ST/ESA/SER.A/427). 2019. Available from: https://population.un.org/wpp/Graphs/1_Demographic%20Profiles/Sub-Saharan%20Africa.pdf. Cited 2022 Jun 2.

[9] Seraphin TP, Joko-Fru WY, Kamaté B, Chokunonga E, Wabinga H, Somdyala NIM (2021). Rising prostate cancer incidence in sub-saharan africa: a trend analysis of data from the African cancer registry network. Cancer Epidemiol Biomarkers Prev.

[10] National Population Commission (NPC). 2006 POPULATION AND HOUSING CENSUS OF THE FEDERAL REPUBLIC OF NIGERIA, Priority Tables. Volume 1. National Population Commission; 2010. Available from: https://nationalpopulation.gov.ng/category/publications/. Cited 2022 Apr 25.

[11] University of Benin Teaching Hospital. University of Benin Teaching Hospital: About Radiotherapy. 2022. Available from: https://ubth.org/clinical-departments/radiotherapy-clinical-oncology/. Cited 2022 Jul 25.

[12] Cancer Registry – UBTH. Available from: https://ubth.org/cancer-registry/. Cited 2022 Apr 25.

[13] Working Group Report (2005). International rules for multiple primary cancers (ICD-0 third edition). Eur J Cancer Prev.

[14] al-Haddad BJS, Jedy-Agba E, Oga E, Ezeome ER, Obiorah CC, Okobia M (2015). Comparability, diagnostic validity and completeness of Nigerian cancer registries. Cancer Epidemiol.

[15] Bray F, Parkin DM (2009). Evaluation of data quality in the cancer registry: principles and methods. Part I: comparability, validity and timeliness. Eur J Cancer.

[16] Parkin DM, Bray F (2009). Evaluation of data quality in the cancer registry: principles and methods Part II. Completeness Eur J Cancer.

[17] Carstensen B, Plummer M, Laara E, Hills M. Epi: A Package for Statistical Analysis in Epidemiology. R package version 2.47. 2022. Available from: https://CRAN.Rproject.org/package=Epi.

[18] National Population Commission. National Population Commission (2006) Population and Housing Census; Priority Table Volume IV, Population Distribution by Age & Sex (State & Local Government Area). Abuja: Federal Republic of Nigeria; 2010. Available from: https://gazettes.africa/archive/ng/2009/ng-government-gazette-dated-2009-02-02-no-2.pdf.

[19] World Bank. World Bank. Nigeria: Population Growth Rate. 2022. Available from: https://data.worldbank.org/share/widget?end=2022&indicators=SP.POP.GROW&locations=NG&start=1961&view=map.

[20] National Primary Health Care Development Agency. Scorecard five report on implementation status of primary health care under one roof (PHCUOR). Abuja, Nigeria; 2019 p. 1–210. Available from: https://nphcda.gov.ng/wp-content/uploads/2022/06/PHCUOR-Scorecard-5-Report.pdf.

[21] Adeloye D, David RA, Aderemi AV, Iseolorunkanmi A, Oyedokun A, Iweala EEJ (2016). An estimate of the incidence of prostate cancer in Africa: a systematic review and meta-analysis. Shore N, editor. PLOS ONE.

[22] Bleyer A, Spreafico F, Barr R (2020). Prostate cancer in young men: an emerging young adult and older adolescent challenge. Cancer.

[23] United Nations D of E and SA Population Division (2019). World Population Prospects 2019: Highlights (ST/ESA/SER.A/423). New York: United Nations; 2019. Available from: http://creativecommons.org/licenses/by/3.0/igo/.

[24] GLOBOCAN 2020. Cancer Today. Estimated number of new cases in 2020, Nigeria, both sexes, ages 0–14 (excl. NMSC). Lyon, France: International Agency for Research on Cancer; 2020. Available from: https://bit.ly/3IwOyam.

[25] Stiller CA, Parkin DM (1996). Geographic and ethnic variations in the incidence of childhood cancer. Br Med Bull.

[26] Raza SA, Jawed I, Zoorob RJ, Salemi JL (2020). Completeness of cancer case ascertainment in international cancer registries: exploring the issue of gender disparities. Front Oncol.

[27] National Population Commission. Civil Registration and Vital Statistics in Nigeria. 2023. Available from: https://nationalpopulation.gov.ng/civil-registration.

